# The Impact of the COVID-19 Pandemic on Food Consumption Behavior: Based on the Perspective of Accounting Data of Chinese Food Enterprises and Economic Theory

**DOI:** 10.3390/nu14061206

**Published:** 2022-03-12

**Authors:** Chung-Cheng Yang, Yahn-Shir Chen, Jianxiong Chen

**Affiliations:** Department of Accounting, National Yunlin University of Science and Technology, Yunlin 64002, Taiwan; ycc@yuntech.edu.tw (C.-C.Y.); chenys@yuntech.edu.tw (Y.-S.C.)

**Keywords:** food consumption behavior, Chinese food enterprises, COVID-19, accounting data, economic theory

## Abstract

Since the outbreak of the COVID-19 pandemic, the rapid spread of COVID-19 around the world has become one of the main focuses of concern in almost every country, and governments have taken numerous measures to prevent/mitigate the spread of the disease. As an essential social determinant, COVID-19 has significantly impacted consumers’ food consumption behavior and healthy eating habits/behaviors. The purpose of this study is to analyze the impact of COVID-19 on food consumption behavior, and the main goal was to assess the possible problems (such as food waste and weight gain) caused by changes in food consumption behavior during the pandemic. Based on the accounting data of Chinese enterprises found in the China Stock Market and Accounting Research (CSMAR) database, this study uses economic theory and the translog function to conduct an average partial effect (APE) analysis of the pandemic, and finds that the COVID-19 pandemic has increased Chinese people’s overall food consumption, and the consumption of food from large food enterprises has increased even more (APE = 0.11 vs. APE = 0.31). This study suggests that food waste and weight gain in the Chinese population may be more severe during the pandemic, and it is necessary to enhance food management and weight management through multiple pathways.

## 1. Introduction

### 1.1. The Impact of the COVID-19 Pandemic on Food Consumption

On 30 January 2020, the World Health Organization (WHO) declared a global health emergency [1], and on 11 March 2020, COVID-19 was declared a global pandemic [2]. The COVID-19 crisis has presented unique challenges [3]; the pandemic has caused one of the worst crises in the world’s economy since the end of World War II [4,5]. COVID-19 has also changed people’s hygiene behaviors [6,7], and affected people’s food choice motive [8,9,10,11,12,13] and appetitive traits [14,15]. The pandemic, and the restrictive measures taken to control the pandemic, have often led to uncertainty in demand and supply disruptions, which have ultimately affected product prices and household food consumption. In addition, containment measures such as isolation, maintaining social distancing, closing businesses, and closing borders have led to a sharp decline in economic activity, affecting household income and consumer behavior [16,17], which is once again reflected in household expenditures. Although food is a basic necessity, nevertheless, food consumption is not immune to this type of crisis. In China, both SARS and COVID-19 have severely affected the food consumption behavior of the Chinese population. During the SARS pandemic, people were worried that the virus would be transmitted through the air, and they generally reduced their frequency of going out and meeting each other. The uncertain economic outlook during these crises had a negative impact on people’s income expectations, consumer spending dropped significantly [18], and access to food was severely affected [19].

In the early stages of the epidemic, people’s food consumption may have declined due to factors such as being unable to work or reduced incomes [20,21], using more savings for medical care [22], and being impacted by fear [23]. These changes are not only reflected in the quantity but also in the quality and type of food consumed—consuming cheaper alternatives [24], reducing the consumption of fruits [25,26], reducing animal-derived foods such as meat and poultry [26], and using more shelf-stable packaged foods [27]. Similarly, during a pandemic, consumers’ dietary patterns and food sources may also change. Consumers may prefer food purchasing channels that provide online ordering and door-to-door delivery, and choose channels that they think are safer and can maintain a higher household inventory to reduce the number of times they go to the market.

Contrary to the view that the pandemic has reduced food consumption, some studies suggest that the pandemic has increased food consumption. Some scholars have found that restrictions imposed in response to the pandemic have led to changes in people’s food consumption behavior, such as an increase in the amount of food purchased [28]. Additionally, disease-related fear may have led to overeating, as stress causes people to increase their food consumption [29].

### 1.2. Hypothesis Development

COVID-19 is impacting many aspects of people’s food consumption behavior. In terms of snacking, many studies have reported significant changes in snacking frequency and behavior during COVID-19 [30,31,32,33,34,35,36,37,38]. The nutritional composition of food is closely related to consumers’ food choice motives [39]. Early in the pandemic, the World Health Organization issued nutritional guidelines recommending the consumption of fresh vegetables, fruits, legumes, and whole grains to obtain adequate dietary fiber [40]. Fruits and vegetables have positive effects on people’s physical [41,42,43] and mental health [41,44,45,46]. Many studies have reported changes in fresh produce consumption in people’s diets during the pandemic [31,32,34,36,47,48,49,50,51,52,53,54]; people’s fruit intake increased during the pandemic [47,49,50,53]. In terms of the number of meals, studies report an increase in the number and frequency of meals people ate during lockdown [32,36,48,49,55]. In addition, according to consumption trends, home cooking has increased during the pandemic [34,51,52]. Many of the above studies have shown that during the pandemic, people’s family cooking frequency and meal frequency generally increased, and different types of food consumption showed an increasing trend.

The COVID-19 pandemic has also had a significant impact on the food e-commerce market; the global food e-commerce market has seen considerable growth during the pandemic, and the growth trend is mainly due to the significant increasing consumer demand during the COVID-19 pandemic. The market is expected to reach USD 66.95 billion in 2025, growing at a CAGR of 21%. At the same time, the increase in smartphone users has also boosted global food e-commerce sales. Smartphone users are the main online buyers in the food industry, and their increasing numbers and Internet penetration are driving the growth of food e-commerce. The growing popularity of e-commerce websites and mobile commerce applications (e.g., mobile online shopping, mobile payment, etc.) for different products has contributed to the increasing popularity of the Internet and the popularity of online food purchases [56,57].

Due to the characteristics and frequency of food purchases, online food purchases are quite different from general online shopping [58,59]. The growing popularity of buying food online is also related to several other reasons. These include changes to people’s lifestyles and consumption habits [60], as well as to the convenience of buying food online. Buying food online saves consumers a lot of time [61]. It also provides several advantages, including the opportunity for consumers to compare more different categories of food products, some of which may not be available in the local market, and the ability for consumers to easily browse a wide variety of food-related information through the Internet (e.g., images, ingredients, and allergen lists, etc.), and the ability to buy food at any time of the day and receive it directly at home, reducing the physical effort to purchase food, saving time and money [62,63,64]. A surprising finding is that after the pandemic began, online food orders in the US increased by 700% in the first quarter of 2020 compared to the same period the previous year [65]. The COVID-19 pandemic has considerably pushed people to hoard food [66], and more and more consumers are buying food online to maintain social distancing. In addition, by purchasing food online, consumers can ensure that they obtain the food they want instead of facing empty shelves. 

In the United States, the top 10 food companies control more than half of food sales [67], and globally, this is around 15% and rising. More than half of the world’s soft drinks are produced by large food companies [68]. Three-quarters of the world’s food sales involve processed foods, with the most prominent manufacturers accounting for more than one-third of the global market [69]. The world food system is not a competitive market for small producers but an oligopoly. In addition, during the pandemic, people generally increased their frequency of shopping online for food and the quantity of food purchased online [33]. Under the pandemic crisis, large food companies are more favored by consumers because of their relatively well-established online sales channels. Based on this, the following hypothesis was formulated:

**Hypothesis** **1.** *The COVID-19 pandemic has increased people’s overall food consumption, and people’s consumption of food from large food enterprises has increased even more*.

### 1.3. Research Goal and Contribution

The main goal of this study is to assess the problems (e.g., food waste and weight gain) that may result from the overall changes in the food consumption behavior of the Chinese population during the pandemic. Many previous studies have also discussed the impact of the COVID-19 pandemic on food consumption. For example, many scholars reported that individual dietary habits changed [70,71,72,73,74,75,76] or remained essentially unchanged [52,77,78] during the pandemic. Di Renzo et al. [52] noted that more than half of Italians consumed the same amount of food during the pandemic as before. During the pandemic, researchers found that in Canada [73], Denmark [74], Poland [75], and the United Kingdom [76], food consumption has increased. Food consumption did not change significantly in the Netherlands [77] and Addis Ababa [78]. The above studies discussed the changes in food consumption of populations in many countries during the pandemic. How did food consumption change in China during the pandemic? To the best of our knowledge, current research on changes in food consumption of the Chinese population during the COVID-19 pandemic is mainly based on different food categories. For example, it has been reported that the consumption of fresh food has increased significantly compared to other fast-moving consumer goods since the COVID-19 outbreak [70]. At the same time, Chinese people increased their consumption of cooked meat products, and consumed more fruits and vegetables [72] and other high-protein foods (e.g., milk) [71], but reduced their consumption of raw and cold foods [72].

However, no previous research has investigated the overall impact of the COVID-19 pandemic on the food consumption behavior of the Chinese population. In contrast to existing studies on food consumption during the pandemic, our study is based on the perspective of Chinese food enterprises and focuses on the overall impact. Through this unique perspective, we assess the overall impact of the COVID-19 pandemic on the food consumption behavior of the Chinese population, filling the current research gap. Other contributions of this paper are as follows: 1. As the largest developing country, China needs to feed 1.4 billion people every year. It is of great significance to study the overall change in the food consumption behavior of the Chinese population during the COVID-19 pandemic to promote the understanding of the overall impact of the COVID-19 pandemic on the food consumption of the Chinese people and solve possible related problems. 2. With the rapid economic development of Asian countries, food waste has also increased rapidly, from 278 million tons to 416 million tons per year, resulting in a corresponding increase in the contribution of Asian countries to greenhouse gas emissions from 8% to 10% [79]. Our research contributes in part to the achievement of the United Nations 2030 Sustainable Development Goal (SDG12.3) [80] to reduce food waste during the pandemic in China.

## 2. Method

### 2.1. Corporate Sales and Food Consumption

Barclay and Brand-Miller [81] used the sales data provided by beverage manufacturers to study people’s consumption behavior, and the results showed that in the past 30 years, the sugar consumption of Australians has declined. Czoli et al. [82] used sales data to estimate the consumption of sugar-sweetened beverages and found that between 2004 and 2015, the consumption of sugar-sweetened beverages by Canadians showed a downward trend. Castaldelli-Maia et al. [83] used alcohol sales data as a proxy for alcohol consumption to assess the impact of increased alcohol consumption among Americans during the pandemic. Similar to previous studies, we use data related to enterprise sales to evaluate people’s food consumption behavior during the pandemic.

From the supply side perspective, the increase in the population’s consumption of food is closely related to the increase in food sales. In basic economic theory, product price multiplied by sales quantity equals revenue. In view of the fact that the Chinese government has strictly controlled food prices during the pandemic, food prices have fluctuated less [84]. In this case, the sales volume of food will directly cause changes in the company’s revenue. From the demand perspective, changes in the amount of food purchased by consumers will directly impact food companies’ revenue. The impact of COVID-19 on food consumption, from the perspective of food companies and accounting statements, will be reflected in the changes in the revenue of the food industry. Therefore, we use the changes in the revenue of the food industry to act as a proxy variable for the changes in China’s food consumption. It should be noted that the food industry in this study is broad, that is, in addition to food in a narrow sense, it also includes agricultural food, which is different from the previous study by Chen and Yang [85].

Sustainable development is closely related to economic efficiency [86]. Economic theory believes that changes in the overall sales and revenue of the industry can effectively reflect changes in consumption trends. Therefore, this study uses the translog function of food enterprises to evaluate the impact of the pandemic on food consumption behavior, to verify our hypothesis: the COVID-19 pandemic has increased people’s overall food consumption, and people’s consumption of food from large food enterprises has increased even more. 

### 2.2. Data and Variables

#### 2.2.1. Data Source and Sample Period 

Different from studies conducted by recruiting subjects, the data of this study are based on the data of Chinese food enterprises provided by the China Stock Market and Accounting Research (CSMAR) database. The CSMAR database is positioned as a research database serving universities and financial institutions for research and quantitative analysis. The CSMAR database refers to the classification standards of CRSP, COMPUSTAT, and other databases, combines the actual situation of the Chinese market, and divides the database into market information, company, economy, stock, green economy, factor research, bond market, fund, bank, and other sub-databases. It covers research-based databases in significant social, economic, and financial fields, such as China’s macro-economy, industry, foreign exchange, and securities. It is one of the essential basic tools for universities and financial institutions to conduct empirical research and investment.

The CSMAR database is the largest database of listed companies in China, through which we obtained the accounting data for this paper, including the total revenue and various input variables of China’s food industry from 2015 to 2020. Taking into account the reasonableness of the data, we removed unreasonable data, such as the food companies whose total revenue was equal to 0, the number of employees of each type was equal to 0, fixed assets were equal to 0, and the amount of R&D investment was equal to 0, and so on. Finally, 487 valid observations were obtained. These 487 valid observations were used to create the model.

#### 2.2.2. Variable Definitions

We defined the variables on the left side of the model as follows: the total revenue of the food industry (*FOREVENUE*) is the dependent variable. The variables on the right side of the model are the various types of inputs in the food industry. On the one hand, since human capital plays a vital role in developing enterprises [85,87,88], we use management employees (*MANAGERS*), research and development employees (*RDS*), and ordinary employees (*ORDINARYS*) as proxy variables to measure personnel input in the food industry. On the other hand, we use the net fixed assets (*FASSETS*) listed in the financial statements of the listed food companies to represent the fixed asset investment, and the research and development investment amount (*DEVEL*) to represent the research and development investment.

We use the COVID-19 pandemic (*COVID19*) as a dummy variable. When *COVID19* is equal to 1, it represents the year 2020; when *COVID19* is equal to 0, it represents the five years from 2015 to 2019. We use this dummy variable to examine the impact of the pandemic on food consumption behavior. Another dummy variable in this study is China’s top ten food companies (*LARGE*), the fact that their food is generally more trusted in the market. In the context of the pandemic, the sales of food produced by *LARGE* and non-*LARGE* enterprises in the market are quite different. We add the top ten food enterprises (*LARGE*) as a dummy variable. If the food company is one of the top ten food companies in China, then *LARGE* is equal to 1; otherwise, it is equal to 0. Table 1 lists the exact meanings of all variables in this study.

## 3. Results

### 3.1. Descriptive Statistics and Correlation Matrix

Table 2 shows the descriptive statistics. The medians of almost all variables are well below the mean (*FOREVENUE*, *MANAGERS*, *RDS*, *ORDINARYS*, *EMPLOYEES*, *FASSETS*, and *DEVEL*), indicating that the data are not normally distributed, but skewed to the right. High standard deviations in descriptive statistics suggest that food enterprises vary widely in composition and size in terms of the distribution of studied variables. From 2015 to 2020, the average value of total revenue (*FOREVENUE*) was 1.027 billion, 1.029 billion, 1.082 billion, 1.162 billion, 1.206 billion, and 1.589 billion, respectively, showing an upward trend. From 2015 to 2020, the average number of managers (*MANAGERS*) was 14.98, 14.36, 14.24, 14.12, 13.97, and 13.89, showing a downward trend. From 2015 to 2020, the average number of research and development personnel (*RDS*) was 177.95, 179.65, 195.38, 204.68, 217.17, and 236.12, and the number was increasing year by year. The average number of other employees (*ORDINARYS*) during 2015-2020 was 5946.21, 5627.84, 5483.24, 5728.88, 5300.63, and 5771.39, showing a wavy trend, but the overall trend was decreasing. During 2015–2020, the average fixed assets (*FASSETS*) were 274 million, 283 million, 280 million, 299 million, 332 million, and 392 million, showing an increasing trend year by year. From 2015 to 2020, the average research and development expenses (*DEVEL*) were 9.83 million, 10.55 million, 11.06 million, 12.97 million, 13.75 million, and 14.08 million, showing an increasing trend by year.

Based on the analysis of the actual situation of China’s food industry, from 2015 to 2020, the scale of China’s food market has gradually expanded, and the average revenue of the food industry has increased year by year, from USD 1.027 billion in 2015 to USD 1.589 billion in 2020. The size of the food market in 2020 was the largest in history. From 2016 to 2019, the growth rate of China’s food market scale was 0.15%, 5.20%, 7.41%, and 3.79%, respectively. However, it is worth noting that in 2020, the growth rate of China’s food market increased significantly, increasing to 31.68%. In terms of human resources input, the average human resources of the food industry in 2015 was 6139. However, in the 2016–2020 period, the average human resources in the food industry were lower than in 2015. In 2020, the figure was just 6,021. From 2015 to 2019, Chinese food companies increased their overall investment in fixed assets. In those four years, the investment in fixed assets increased by 21.26%, from USD 274 million to USD 332 million. In terms of R&D, it increased from USD 9.83 million to USD 13.75 million, and the R&D investment in 2019 increased by 39.88% compared to 2015, which fully demonstrates that the Chinese food market attaches particular importance to the research and development of new foods. In 2020, despite the adverse effects of the pandemic, Chinese food companies continued to increase their investment in *RDS*, *ORDINARYS*, *FASSETS*, and *DEVEL*, which shows that Chinese food companies still generally have tremendous confidence in people’s food consumption.

Table 3 is the Pearson and Spearman correlation coefficient table of this study. The correlation coefficient between *COVID19* and *FOREVENUE* is positive. Meanwhile, combined with Table 2, we can see that under the pandemic of 2020, the revenue of the food industry still is trending upward. However, it should be noted that the correlation coefficient only represents a one-to-one relationship between variables, and the relationship does not consider the influence of other variables. In econometrics, the impact of other variables must be added for a comprehensive analysis. Therefore, in the next section, we will explore the impact of the COVID-19 pandemic on food consumption behavior through the translog revenue function.

### 3.2. Translog Model Estimates 

#### 3.2.1. Model Test

Please refer to Appendix A for the model derivation process. The resulting data in Table 4 and Table 5 were used to verify the model. In order to better reflect the study results, we used the translog revenue function in this study. Compared with the Cobb–Douglas (log-linear) function, many advantages of the translog revenue function make it more suitable for this study. Table 4 shows the empirical results of this study. However, this study needs to determine whether the translog revenue function can accurately describe the results. Therefore, we need to test model as follows:

(1) $$\alpha_{il}=\beta_{11}=\delta_{11}=\gamma_{i1}=\varepsilon_{i1}=\theta_{11}=0$$

In the above equation, i=1, 2, 3. As shown in Table 4, the *F*-statistic of this model is equal to 5.07. This test result indicates that compared to the Cobb–Douglas function, the translog revenue function is more suitable for estimating the impact of the pandemic on the food market.

#### 3.2.2. Food Consumption Behavior during the COVID-19 Pandemic

As shown in Table 5, the APE of *COVID19* on the *FOREVENUE* is positive. In addition, in Table 4, the coefficient of translog for *COVID19* is also significantly positive. From this, we can see that during the COVID-19 pandemic in China, there was an increase in people’s overall food consumption. In addition, one of the most critical impacts of the pandemic on food consumption behavior is that more consumers have chosen to buy food online. We expect that even if the pandemic eases in the future, buying food online will continue as a trend. Table 5 shows that the APE of *COVID19* for non-*LARGE* (0.113) is smaller than the APE of *COVID19* for *LARGE* (0.311), indicating that Chinese people have been more inclined to buy food produced by large enterprises during the pandemic. This verifies the hypothesis of this study. The COVID-19 pandemic has had a significant economic impact on SMEs [89], and the lack of online sales channels for SMEs has directly affected consumer choices.

## 4. Discussion

Since 2020, the COVID-19 pandemic has affected consumers’ consumption habits and food purchasing behavior [24,25,26,51,73,74,75,76]. Lockdowns, social distancing, business closures, and people’s insecurity have led to a sharp decline in economic activity [4,5], affecting household income and expenditures [20,21]. During the pandemic, restaurants in many countries and regions were forced to close, and people were spending considerably more time at home. The COVID-19 pandemic has also caused serious setbacks in the global economy, and unemployment rates in most countries have risen sharply. Although more countries restarted their economies after the summer of 2020, the crisis of disappearing jobs has not been alleviated. The International Labour Organization [90] reported that global working hours were reduced by 14% in the second quarter of 2020 alone, which is equivalent to the disappearance of 400 million full-time jobs that work 48 h a week. Compared with the previously released monitoring results, global working hours continued to drop by 10.4%. The pandemic has made it impossible for many people around the world to go to work, which could reduce food consumption.

During the pandemic, people have consciously reduced going out for personal safety. From the perspective of consumer behavior, the pandemic has prevented people from going to the market to buy food to a certain extent. Moreover, around the world, the reduction in income and uncertainty about future income caused by the pandemic has made people consciously reduce their expenditures on many foods, and has moved people to save more to cope with the risk of economic uncertainty during the pandemic. Some studies reported that in Ethiopia [91] and Guatemala [92], the food consumer markets have been negatively affected by COVID-19. Our findings are quite different from those of Tesfaye et al. [91] and Ceballos et al. [92], but our findings are similar to those of Sim et al. [28] and Di Crosta et al. [93]. Sim et al. [28] reported panic buying of food due to the spread of the pandemic. Di Crosta et al. [93] found that the COVID-19 pandemic increased consumers’ psychological need to purchase essential and non-essential products. Based on food-related data in China and the translog function, this study found that the COVID-19 pandemic has increased people’s overall food consumption. 

The possible reasons for this result are as follows. First, due to the strong driving force of the Chinese economy and the extremely high level of savings of the Chinese population, soon after the outbreak of the pandemic, people’s overall willingness to consume quickly recovered, which had a positive effect on the sales of food in China. The Chinese Consumer Attitude Survey [94] shows that with reducing the negative impact of the pandemic in China, Chinese consumer confidence is gradually recovering. This means that most consumers’ spending on consumption will return to a relatively high level. Johnny et al. [94] conducted a questionnaire survey on the consumption of consumer products with approximately 2500 Chinese consumers. Most interviewees indicated that the use of most consumer products will return to pre-pandemic levels. Moreover, 60~70% of the interviewees expected that the consumption of consumer products will return to normal levels or slightly increase; 10% of the interviewees said that the consumption of consumer products will be greatly increased, which may reflect a delay in demand to some extent. Only 20~30% of the interviewees said that they would continue to be cautious and reduce the consumption of various consumer products slightly or substantially. Second, in response to the possible negative impact of the pandemic, Chinese food companies have generally taken positive actions to increase marketing and promotion efforts to encourage consumption recovery.

However, we have two concerns about the findings of the study. First, people spent less time outdoors during the pandemic than before, and in this context, the surge in food purchases is likely to exacerbate food waste. Food waste is a critical economic and environmental issue; taking the European Union as an example, it wastes around 88 million tons of food every year, and the associated cost is estimated at EUR 143 billion. Notably, households produced an enormous amount of food waste, accounting for more than 53% of food waste in the European Union [95]. Furthermore, food waste is also an ethical issue as more than 820 million people worldwide are undernourished, and many more eat low-quality food, leading to micronutrient deficiencies and other nutritional problems [96]. Second, the longer the relevant government measures to control the pandemic persist, the higher the likelihood that increased food consumption will increase obesity prevalence and related diseases [97]. With the ongoing pandemic, severe long-term health consequences are expected. This study believes that, in China, the food needs of the majority of the population are adequately secured after the pandemic outbreak. However, enhanced food management and weight management are important issues to focus on during the pandemic.

The total observations of this study are 487, with 383 records in 2015–2019 and only 104 records in 2020. It is undeniable that the study would have obtained more accurate results if more years of observations were available after the outbreak of the COVID-19 pandemic. However, regarding the data after the outbreak of the COVID-19 pandemic, presently, any researcher can only obtain the data for 2020. This is because, in China, all companies’ 2021 annual financial statements will usually only be disclosed after April 2022. For example, Zhongjing Food Company [98], Joyvio Food Company [99], and Sanquan Food Company [100] have announced that the 2021 annual financial statement disclosure times will be 23 April 2022, 8 April 2022, and 15 April 2022, respectively. In other words, the current observations in this study are already the most accurate results given the current data available. 

This study is limited by the availability of data. First, our data does not cover changes in food markets and food consumption behavior since January 2021. Second, this study has only 104 records for 2020 (time during COVID-19 pandemic), and all others are for the period before the COVID-19 pandemic, which is also one of the limitations of this study. 

## 5. Conclusions

Based on food-related accounting data in China and economic theory, this study found that the COVID-19 pandemic has increased people’s overall food consumption in China. Although the overall consumption of food in China has increased due to the COVID-19 pandemic, we also need to face some societal challenges brought about by the pandemic. Any major crisis will severely hit the bottom of the income pyramid and reduce the nutritional intake of this part of the population. Therefore, even when the overall consumption of food in society rises, the low-income groups in China who are most vulnerable to the pandemic may face difficulties in consuming sufficient food. Government support is urgently needed to ensure the nutritional security of the poorer part of the population.

## Figures and Tables

**Table 1:** Variable definitions.

Variable		Definition
Theoretical Variable	Proxy Variable	
*r*	*FOREVENUE*	Total revenue of food enterprise
$x_{it}^{m}$	*MANAGER* *S*	Total number of management personnel
$x_{it}^{r}$	*RDS*	Total number of research and development personnel
$x_{it}^{o}$	*ORDINARY* *S*	Total number of ordinary personnel
	*EMPLOYEES*	Total number of employees
$x_{it}^{a}$	*FASSET* *S*	Net fixed assets
$x_{it}^{d}$	*DEVEL*	R&D investment amount
	*LARGE*	If the food enterprise is not the top ten food enterprises, *LARGE* is equal to 0; otherwise, it is equal to 1
	*COVID19*	If the year is 2015–2019, *COVID19* is equal to 0; otherwise, it is equal to 1

**Table 2:** Changes in variables from 2015 to 2020.

	**2015 (*n* = 62)**						**2016 (*n* = 69)**					
**Variables**	**Mean**	**Median**	**Max**	**Min**	**Std**	**Kurt**	**Mean**	**Median**	**Max**	**Min**	**Std**	**Kurt**
*FOREVENUE*	$1027.17	$324.04	$9673.95	$23.75	$1934.79	14.23	$1028.74	$349.21	$9579.57	$40.74	$1934.79	14.23
*MANAGERS*	14.98	15.00	21.00	10.00	2.44	2.85	14.36	14.00	20.00	10.00	2.27	2.68
*RDS*	177.95	90.50	1596.00	3.00	254.98	18.12	179.65	91.00	1630.00	7.00	254.32	17.61
*ORDINARYS*	5946.21	2424.00	57,606.00	269.00	11,773.47	15.74	5627.84	2325.00	60,145.00	286.00	11189.65	17.70
*EMPLOYEES*	6139.15	2680.50	57,971.00	328.00	11,867.11	15.51	5821.86	2457.00	60,602.00	309.00	11282.64	17.39
*FASSETS*	$273.70	$116.87	$2296.58	$7.57	$438.87	12.97	$283.14	$132.92	$2060.63	$6.69	$426.28	10.02
*DEVEL*	$9.83	$4.17	$79.28	$0.06	$15.73	10.93	$10.55	$4.87	$82.58	$0.07	$16.36	9.81
	**2017 (*n* = 79)**						**2018 (*n* = 81)**					
**Variables**	**Mean**	**Median**	**Max**	**Min**	**Std**	**Kurt**	**Mean**	**Median**	**Max**	**Min**	**Std**	**Kurt**
*FOREVENUE*	$1082.22	$418.42	$10,617.75	$39.95	$1950.52	14.91	$1162.45	$449.88	$12,426.70	$50.18	$2139.28	16.71
*MANAGERS*	14.24	14.00	20.00	9.00	2.34	2.83	14.12	14.00	20.00	9.00	2.52	2.50
*RDS*	195.38	98.00	1635.00	6.00	273.49	16.33	204.68	113.00	1298.00	11.00	250.91	11.18
*ORDINARYS*	5483.24	2391.00	60,851.00	282.00	10,670.69	19.89	5728.88	2350.00	61,437.00	315.00	10,836.65	19.56
*EMPLOYEES*	5692.86	2510.00	61,318.00	301.00	10,768.02	19.45	5947.68	2525.00	61,918.00	359.00	10,926.29	19.18
*FASSETS*	$279.99	$129.46	$2092.09	$11.01	$427.86	10.89	$298.87	$144.09	$2642.64	$10.70	$470.33	14.05
*DEVEL*	$11.06	$3.96	$82.74	$0.03	$17.93	9.81	$12.97	$5.34	$94.07	$0.05	$20.29	8.64
	**2019 (*n* = 92)**						**2020 (*n* = 104)**					
**Variables**	**Mean**	**Median**	**Max**	**Min**	**Std**	**Kurt**	**Mean**	**Median**	**Max**	**Min**	**Std**	**Kurt**
*FOREVENUE*	$1206.49	$385.39	$14,157.00	$36.81	$2375.23	18.60	$1588.73	$408.98	$30,673.50	$19.51	$3979.69	32.36
*MANAGERS*	13.97	14.00	21.00	8.00	2.85	2.56	13.89	14.00	22.00	9.00	2.65	3.22
*RDS*	217.17	108.50	2405.00	7.00	336.40	24.01	236.12	120.50	2652.00	6.00	427.37	23.08
*ORDINARYS*	5300.63	2292.50	69,871.00	268.00	10,503.66	25.21	5771.39	2216.50	94965.00	128.00	12,152.72	33.29
*EMPLOYEES*	5531.77	2414.50	70,600.00	318.00	10,639.71	24.57	6021.39	2394.50	95993.00	172.00	12,334.79	32.43
*FASSETS*	$331.90	$145.03	$3853.85	$7.33	$588.30	20.00	$391.68	$137.32	$4687.54	$7.71	$847.85	18.75
*DEVEL*	$13.75	$5.63	$157.30	$0.10	$24.54	16.69	$14.08	$5.59	$163.59	$0.17	$25.48	16.19

Note: *FOREVENUE*, *FASSETS*, and *DEVEL* are expressed in millions of US dollars. Please see Table 1 for variable definitions.

**Table 3:** Pearson and Spearman correlation coefficients between variables in this study (*p*-values in parentheses).

	(1)	(2)	(3)	(4)	(5)	(6)	(7)	(8)	(9)
(1) *FOREVENUE*	1.000	0.198	0.476	0.785	0.788	0.786	0.557	0.562	0.023
	-----	(0.000)	(0.000)	(0.000)	(0.000)	(0.000)	(0.000)	(0.000)	(0.611)
(2) *MANAGERS*	0.181	1.000	0.179	0.135	0.138	0.180	0.175	0.146	−0.072
	(0.000)	-----	(0.000)	(0.003)	(0.002)	(0.000)	(0.000)	(0.001)	(0.115)
(3) *RDS*	0.404	0.101	1.000	0.483	0.516	0.484	0.729	0.408	0.026
	(0.000)	(0.026)	-----	(0.000)	(0.000)	(0.000)	(0.000)	(0.000)	(0.573)
(4) *ORDINARYS*	0.852	0.204	0.368	1.000	0.999	0.726	0.504	0.523	−0.008
	(0.000)	(0.000)	(0.000)	-----	(0.000)	(0.000)	(0.000)	(0.000)	(0.854)
(5) *EMPLOYEES*	0.854	0.205	0.392	1.000	1.000	0.732	0.527	0.524	−0.007
	(0.000)	(0.000)	(0.000)	(0.000)	-----	(0.000)	(0.000)	(0.000)	(0.871)
(6) *FASSETS*	0.839	0.171	0.528	0.782	0.788	1.000	0.540	0.535	−0.004
	(0.000)	(0.000)	(0.000)	(0.000)	(0.000)	-----	(0.000)	(0.000)	(0.924)
(7) *DEVEL*	0.525	0.187	0.719	0.509	0.524	0.723	1.000	0.408	0.044
	(0.000)	(0.000)	(0.000)	(0.000)	(0.000)	(0.000)	-----	(0.000)	(0.333)
(8) *LARGE*	0.719	0.143	0.503	0.666	0.673	0.667	0.560	1.000	−0.043
	(0.000)	(0.002)	(0.000)	(0.000)	(0.000)	(0.000)	(0.000)	-----	(0.345)
(9) *COVID19*	0.075	−0.064	0.051	0.007	0.008	0.068	0.044	−0.043	1.000
	(0.098)	(0.159)	(0.263)	(0.885)	(0.862)	(0.136)	(0.330)	(0.345)	-----

Note: Pearson coefficient on the left, Spearman coefficient on the right. Please see Table 1 for variable definitions.

**Table 4:** Coefficient estimates for variables in this study (*t*–statistics in parentheses).

Variables	Coefficient	Variables	Coefficient
	*t*-Statistic		*t*-Statistic
Intercept	−20.425	(ln *MANAGERS*) (ln *ORDINARYS*)	−0.113
	(−1.572)		(−0.491)
ln *MANAGERS*	−0.926	(ln *RDS*) (ln *ORDINARYS*)	−0.028
	(−0.213)		(−0.118)
ln *RDS*	−1.755 *	(ln *MANAGERS*)(ln *FASSETS*)	−0.134
	(−1.890)		(−0.760)
ln *ORDINARYS*	−1.192	(ln *RDS*)(ln *FASSETS*)	0.055
	(−1.077)		(0.928)
ln *FASSETS*	3.095 **	(ln *ORDINARYS*)(ln *FASSETS*)	0.027
	(2.481)		(0.543)
ln *DEVEL*	1.569 **	(ln *MANAGERS*) (ln *DEVEL*)	0.071 **
	(2.202)		(2.449)
(ln *MANAGERS*) ^2^	0.823	(ln *RDS*) (ln *DEVEL*)	0.213 ***
	(1.148)		(3.181)
(ln *RDS*) ^2^	−0.087 ***	(ln *ORDINARYS*)(ln *DEVEL*)	−0.020
	(−2.711)		(−0.500)
(ln *ORDINARYS*) ^2^	−0.144 ***	(ln *FASSETS*)(ln *DEVEL*)	−0.154 ***
	(−3.709)		(−4.126)
(ln *FASSETS*) ^2^	−0.045	*LARGE*	0.822 ***
	(−1.183)		(6.905)
(ln *DEVEL*) ^2^	0.056 ***	*COVID19*	0.113 *
	(3.819)		(1.832)
(ln *MANAGERS*)(ln *RDS*)	0.099	*LARGE* *COVID19*	0.198
	(0.502)		(0.987)
Adjusted R–squared	0.834		
Observations	487		
$\mathrm{Test}\mathrm{of}\log–\mathrm{linear}\mathrm{specification}\;(H_{0}:\;\alpha_{il}=\beta_{11}=\delta_{11}=\gamma_{i1}=\varepsilon_{i1}=\theta_{11}=0;\;i=1,\;2,\;3$)			
*F*-statistic	5.07		
Significance level	0.000		

Note: ***, **, and * indicate significance at the 1%, 5%, and 10% level, respectively. Please see Table 1 for variable definitions. ^2^—represents the meaning of square.

**Table 5:** APE of variables.

APE	APE Estimated Value	Significance Test
		$H_{0}:\alpha_{2}=\alpha_{22}=\alpha_{12}=\alpha_{23}=\gamma_{21}=\varepsilon_{21}=0$
*APE_ORDINARYS*	0.551	*F*-statistic = 41.39
		Significance level = 0.00
		$H_{0}:\alpha_{1}=\alpha_{11}=\alpha_{12}=\alpha_{13}=\gamma_{11}=\varepsilon_{11}=0$
*APE_RDS*	−0.083	*F*-statistic = 2.18
		Significance level = 0.04
		$H_{0}:\alpha_{3}=\alpha_{33}=\alpha_{13}=\alpha_{23}=\gamma_{31}=\varepsilon_{31}=0$
*APE_MANAGERS*	0.238	*F*-statistic = 0.76
		Significance level = 0.60
		$H_{0}:\beta_{1}=\beta_{11}=\gamma_{11}=\gamma_{21}=\gamma_{31}=\theta_{11}=0$
*APE_FASSETS*	0.312	*F*-statistic = 17.82
		Significance level = 0.00
		$H_{0}:\delta_{1}=\delta_{11}=\varepsilon_{11}=\varepsilon_{21}=\varepsilon_{31}=\theta_{11}=0$
*APE_DEVEL*	0.137	*F*-statistic = 8.43
		Significance level=0.00
*APE_ LARGE*		$H_{0}:\varphi_{1}=\varphi_{3}=0$
When *COVID19* = 0	0.822	*F*-statistic = 26.50
When *COVID19* = 1	1.020	Significance level=0.00
*APE_COVID19*		$H_{0}:\varphi_{2}=\varphi_{3}=0$
When *LARGE* = 0	0.113	*F*-statistic = 2.97
When *LARGE* = 1	0.311	Significance level = 0.05

## Data Availability

All relevant data are within the manuscript.

## Appendix A

The production function:

(A1) $$y_{it}^{f}=f\left(x_{it}^{m},x_{it}^{o},x_{it}^{r},x_{it}^{a},\;x_{it}^{d}\right)$$

The revenue function of the food enterprises:

(A2) $$\begin{array}{l} \;\;\;r\left(p_{it}^{f};x_{it}^{m},x_{it}^{o},x_{it}^{r},x_{it}^{a},\;x_{it}^{d}\right)=max\;p_{it}^{f}y_{it}^{f}\\ \mathrm{subject}\mathrm{to}y_{it}^{f}=f\left(x_{it}^{m},x_{it}^{o},x_{it}^{r},x_{it}^{a},\;x_{it}^{d}\right)\; \end{array}$$

The converted revenue function of the food enterprises:

(A3) $$\ln r=\alpha_{0}+\delta \ln p_{it}^{f}+\overset{3}{\underset{i=1}{\sum }}\alpha_{i}\ln x_{it}^{e}+\beta_{1}\ln x_{it}^{a}+\delta_{1}ln\;x_{it}^{d}$$

We normalize the above model by $p_{it}^{f}$*=1*:

(A4) $$\ln r=\alpha_{0}+\overset{3}{\underset{i=1}{\sum }}\alpha_{i}\ln x_{it}^{e}+\beta_{1}\ln x_{it}^{a}+\delta_{1}ln\;x_{it}^{d}$$

Specify the function model:

(A5) $$\begin{array}{c} ln\;r=\alpha_{0}+\overset{3}{\underset{i=1}{\sum }}\alpha_{i}\;ln\;x_{it}^{e}+\beta_{1}ln\;x_{it}^{a}+\delta_{1}ln\;x_{it}^{d}+\frac{1}{2}\overset{3}{\underset{i=1}{\sum }}\overset{3}{\underset{l=1}{\sum }}\alpha_{il}\;ln\;x_{it}^{e}\;ln\;x_{it}^{l}+\frac{1}{2}\beta_{11}{\left(\ln x_{it}^{a}\right)}^{2}+\frac{1}{2}\delta_{11}{\left(\ln x_{it}^{d}\right)}^{2}\\ +\overset{3}{\underset{i=1}{\sum }}\gamma_{i1}ln\;x_{it}^{e}\;ln\;x_{it}^{a}+\overset{3}{\underset{i=1}{\sum }}\varepsilon_{i1}ln\;x_{it}^{e}\;ln\;x_{it}^{d}+\theta_{11}ln\;x_{it}^{a}\;ln\;x_{it}^{d} \end{array}$$

We rewrite the above equation:

(A6) $$\begin{array}{ll} \ln FOREVENUE & =\alpha_{0}+\alpha_{1}\ln MANAGERS+\alpha_{2}\ln RDS+\alpha_{3}\ln ORDINARYS+\beta_{1}\ln FASSETS+\delta_{1}\ln DEVEL\\ & +\frac{1}{2}\;\alpha_{11}\;{\left(ln\;MANAGERS\right)}^{2}+\frac{1}{2}\;\alpha_{22}\;{\left(ln\;RDS\right)}^{2}+\frac{1}{2}\;\alpha_{33}\;{\left(ln\;ORDINARYS\right)}^{2}\\ & +\frac{1}{2}\;\beta_{11}\;{\left(ln\;FASSETS\right)}^{2}+\frac{1}{2}\;\delta_{11}\;{\left(ln\;DEVEL\right)}^{2}+\alpha_{12}\ln MANAGERS\ln RDS\\ & +\alpha_{13}\ln MANAGERS\ln ORDINARYS+\alpha_{23}\;ln\;RDS\;ln\;ORDINARYS\\ & +\gamma_{11}\ln MANAGERS\ln FASSETS+\gamma_{21}\;ln\;RDS\;ln\;FASSETS\\ & +\gamma_{31}\;ln\;ORDINARYS\;ln\;FASSETS+\varepsilon_{11}\ln MANAGERS\ln DEVEL+\varepsilon_{21}ln\;RDS\;\;ln\;DEVEL\\ & +\varepsilon_{31}\;ln\;ORDINARYS\;ln\;DEVEL+\theta_{11}\;ln\;FASSETS\;ln\;DEVEL+\varphi_{1}\;LARGE+\varphi_{2}\;COVID19\\ & +\varphi_{3}\;LARGE\;COVID19 \end{array}$$

The APE of *MANAGERS* on *FOREVENUE**:*

(A7) $$\begin{array}{l} \partial \hat{\;ln\;FOREVENUE}/\partial \;ln\;MANAGERS\\ \;\;\;\;\;\;\;\;={\hat{\alpha }}_{1}+{\hat{\alpha }}_{11}\;\bar{ln\;MANAGERS}+{\hat{\alpha }}_{12}\;\bar{ln\;RDS}+{\hat{\alpha }}_{13}\;\bar{ln\;ORDINARYS}\\ \;\;\;\;\;\;\;\;+{\hat{\gamma }}_{11}\;\bar{ln\;FASSETS}+{\hat{\varepsilon }}_{11}\;\bar{ln\;DEVEL}\; \end{array}$$

The APE of *RDS* on *FOREVENUE**:*

(A8) $$\begin{array}{l} \partial \hat{\;ln\;FOREVENUE}/\partial \;ln\;RDS\\ \;\;\;\;\;\;\;\;={\hat{\alpha }}_{2}+{\hat{\alpha }}_{22}\;\bar{ln\;RDS}+{\hat{\alpha }}_{12}\;\bar{ln\;MANAGERS}+{\hat{\alpha }}_{23}\;\bar{lnORDINARYS}\\ \;\;\;\;\;\;\;\;+{\hat{\gamma }}_{21}\;\bar{ln\;FASSETS}+{\hat{\varepsilon }}_{21}\;\bar{ln\;DEVEL} \end{array}$$

The APE of *ORDINARYS* on *FOREVENUE**:*

(A9) $$\begin{array}{l} \partial \hat{\;ln\;FOREVENUE}/\partial \;ln\;ORDINARYS\\ \;\;\;\;\;\;\;\;={\hat{\alpha }}_{3}+{\hat{\alpha }}_{33}\;\bar{ln\;ORDINARYS}+{\hat{\alpha }}_{13}\;\bar{ln\;MANAGERS}+{\hat{\alpha }}_{23}\;\bar{ln\;RDS}\\ \;\;\;\;\;\;\;\;+{\hat{\gamma }}_{31}\;\bar{ln\;FASSETS}+{\hat{\varepsilon }}_{31}\;\bar{ln\;DEVEL} \end{array}$$

The APE of *FASSETS* on *FOREVENUE**:*

(A10) $$\begin{array}{l} \partial \hat{\;ln\;FOREVENUE}/\partial \;ln\;FASSETS\\ \;\;\;\;\;\;\;\;={\hat{\beta }}_{1}+{\hat{\beta }}_{11}\;\bar{ln\;FASSETS}+{\hat{\gamma }}_{11}\;\bar{ln\;MANAGERS}+{\hat{\gamma }}_{21}\;\bar{ln\;RDS}\\ \;\;\;\;\;\;\;\;+{\hat{\gamma }}_{31}\;\bar{ln\;ORDINARYS}+{\hat{\theta }}_{11}\bar{ln\;DEVEL} \end{array}$$

The APE of *DEVEL* on *FOREVENUE**:*

(A11) $$\begin{array}{l} \partial \hat{\;ln\;FOREVENUE}/\partial \;ln\;DEVEL\\ \;\;\;\;\;\;\;\;={\hat{\delta }}_{1}+{\hat{\delta }}_{11}\;\bar{ln\;DEVEL}+{\hat{\varepsilon }}_{11}\;\bar{ln\;MANAGERS}+{\hat{\varepsilon }}_{21}\;\bar{ln\;RDS}\\ \;\;\;\;\;\;\;\;+{\hat{\varepsilon }}_{31}\;\bar{ln\;ORDINARYS}+{\hat{\theta }}_{11}\;\bar{ln\;FASSETS} \end{array}$$

The APE of *LARGE* on *FOREVENUE**:*

(A12) $$\partial \hat{\;ln\;FOREVENUE}/\partial \;ln\;LARGE=\varphi_{1}+\varphi_{3}\;COVID19$$

The APE of *COVID19* on *FOREVENUE*:

(A13) $$\partial \hat{\;ln\;FOREVENUE}/\partial \;COVID19=\varphi_{2}+\varphi_{3}\;LARGE$$

## References

[1] Velavan T.P., Meyer C.G. (2020). The COVID-19 epidemic. Trop. Med. Int. Health.

[2] World Health Organization (2020). WHO Director-General’s Opening Remarks at the Media Briefing on COVID-19—11 March. https://www.who.int/director-general/speeches/detail/who-director-general-s-opening-remarks-at-the-media-briefing-on-covid-19---11-march-2020.

[3] Borio C. (2020). The COVID-19 economic crisis: Dangerously unique. Bus. Econ..

[4] Gautam S., Hens L. (2020). COVID-19: Impact by and on the environment, health and economy. Environ. Dev. Sustain..

[5] Adams-Prassl A., Cloyne J., Dias M.C., Parey M., Ziliak J.P. (2020). The COVID-19 Economic Crisis. Fisc. Stud..

[6] Guzek D., Skolmowska D., Głąbska D. (2020). Analysis of Gender-Dependent Personal Protective Behaviors in a National Sample: Polish Adolescents’ COVID-19 Experience (PLACE-19) Study. Int. J. Environ. Res. Public Health.

[7] Skolmowska D., Głąbska D., Guzek D. (2020). Hand Hygiene Behaviors in a Representative Sample of Polish Adolescents in Regions Stratified by COVID-19 Morbidity and by Confounding Variables (PLACE-19 Study): Is There Any Association?. Pathogens.

[8] Sorić T., Brodić I., Mertens E., Sagastume D., Dolanc I., Jonjić A., Delale E.A., Mavar M., Missoni S., Peñalvo J.L. (2021). Evaluation of the Food Choice Motives before and during the COVID-19 Pandemic: A Cross-Sectional Study of 1232 Adults from Croatia. Nutrients.

[9] Głąbska D., Skolmowska D., Guzek D. (2020). Population-Based Study of the Changes in the Food Choice Determinants of Secondary School Students: Polish Adolescents’ COVID-19 Experience (PLACE-19) Study. Nutrients.

[10] Martínez-Vázquez S.E., Ceballos-Rasgado M., Posada-Velázquez R., Hunot-Alexander C., Nava-González E.J., Ramírez-Silva I., Aguilar-López D.K., Quiroz-Olguín G., López-Jara B., Delgado-De-La-Cruz C. (2021). Perceived Diet Quality, Eating Behaviour, and Lifestyle Changes in a Mexican Population with Internet Access during Confinement for the COVID-19 Pandemic: ESCAN-COVID19Mx Survey. Nutrients.

[11] Skolmowska D., Głąbska D., Guzek D. (2021). Differences in Adolescents’ Food Habits Checklist (AFHC) Scores before and during Pandemic in a Population-Based Sample: Polish Adolescents’ COVID-19 Experience (PLACE-19) Study. Nutrients.

[12] Kołota A., Głąbska D. (2021). Analysis of Food Habits during Pandemic in a Polish Population-Based Sample of Primary School Adolescents: Diet and Activity of Youth during COVID-19 (DAY-19) Study. Nutrients.

[13] Głąbska D., Skolmowska D., Guzek D. (2021). Food Preferences and Food Choice Determinants in a Polish Adolescents’ COVID-19 Experience (PLACE-19) Study. Nutrients.

[14] Guzek D., Skolmowska D., Głąbska D. (2020). Appetitive Traits in a Population-Based Study of Polish Adolescents within the PLACE-19 Study: Validation of the Adult Eating Behavior Questionnaire. Nutrients.

[15] Coakley K.E., Le H., Silva S.R., Wilks A. (2021). Anxiety is associated with appetitive traits in university students during the COVID-19 pandemic. Nutr. J..

[16] Roll S., Chun Y., Kondratjeva O., Despard M., Schwartz-Tayri T.M., Grinstein-Weiss M. (2022). Household Spending Patterns and Hardships during COVID-19: A Comparative Study of the U.S. and Israel. J. Fam. Econ. Issues.

[17] Vázquez-Martínez U.J., Morales-Mediano J., Leal-Rodríguez A.L. (2021). The impact of the COVID-19 crisis on consumer purchasing motivation and behavior. Eur. Res. Manag. Bus. Econ..

[18] Zeng C.Y., Feng Y. (2004). Viewing of the strength of the national economy for fighting against crisis from the case of SRAS. Beijing Inst. Technol..

[19] Wen Z., Huimin G., Kavanaugh R.R. (2005). The Impacts of SARS on the Consumer Behaviour of Chinese Domestic Tourists. Curr. Issues Tour..

[20] Su C.-W., Dai K., Ullah S., Andlib Z. (2021). COVID-19 pandemic and unemployment dynamics in European economies. Econ.Res.-Ekonom. Istraž..

[21] Midões C., Seré M. (2021). Living with Reduced Income: An Analysis of Household Financial Vulnerability Under COVID-19. Soc. Indic. Res..

[22] Vaidheeswaran S., Karmugilan M.K. (2021). Consumer buying behaviour on healthcare products and medical devices during COVID-19 pandemic period-a new spotlight. NVEO.

[23] Immordino G., Jappelli T., Oliviero T., Zazzaro A. (2021). Fear of COVID-19 contagion and consumption: Evidence from a survey of Italian households. Health Econ..

[24] Chang Y.Y.-C., Wu P.-L., Chiou W.-B. (2021). Thoughts of social distancing experiences affect food intake and hypothetical binge eating: Implications for people in home quarantine during COVID-19. Soc. Sci. Med..

[25] Litton M., Beavers A. (2021). Food Insecurity is Associated with Reducing Fruit and Vegetable Intake During COVID-19. J. Acad. Nutr. Diet..

[26] Jia P., Liu L., Xie X., Yuan C., Chen H., Guo B., Zhou J., Yang S. (2020). Changes in dietary patterns among youths in China during COVID-19 epidemic: The COVID-19 impact on lifestyle change survey (COINLICS). Appetite.

[27] Maffoni S., Brazzo S., De Giuseppe R., Biino G., Vietti I., Pallavicini C., Cena H. (2021). Lifestyle Changes and Body Mass Index during COVID-19 Pandemic Lockdown: An Italian Online-Survey. Nutrients.

[28] Sim K., Chua H.C., Vieta E., Fernandez G. (2020). The anatomy of panic buying related to the current COVID-19 pandemic. Psychiatry Res..

[29] Razzoli M., Pearson C., Crow S., Bartolomucci A. (2017). Stress, overeating, and obesity: Insights from human studies and preclinical models. Neurosci. Biobehav. Rev..

[30] Bakhsh M.A., Khawandanah J., Naaman R.K., Alashmali S. (2021). The impact of COVID-19 quarantine on dietary habits and physical activity in Saudi Arabia: A cross-sectional study. BMC Public Health.

[31] Ammar A., Brach M., Trabelsi K., Chtourou H., Boukhris O., Masmoudi L., Bouaziz B., Bentlage E., How D., Ahmed M. (2020). Effects of COVID-19 Home Confinement on Eating Behaviour and Physical Activity: Results of the ECLB-COVID19 International Online Survey. Nutrients.

[32] Scarmozzino F., Visioli F. (2020). COVID-19 and the Subsequent Lockdown Modified Dietary Habits of Almost Half the Population in an Italian Sample. Foods.

[33] Almughamis N., Alasfour S., Mehmood S. (2020). Poor eating habits and predictors of weight gain during the COVID-19 quarantine measures in Kuwait: A cross sectional study. F1000Research.

[34] Deschasaux-Tanguy M., Druesne-Pecollo N., Esseddik Y., de Edelenyi F.S., Allès B., Andreeva V.A., Baudry J., Charreire H., Deschamps V., Egnell M. (2021). Diet and physical activity during the coronavirus disease 2019 (COVID-19) lockdown (March–May 2020): Results from the French NutriNet-Santé cohort study. Am. J. Clin. Nutr..

[35] Gallo L.A., Gallo T.F., Young S.L., Moritz K.M., Akison L.K. (2020). The Impact of Isolation Measures Due to COVID-19 on Energy Intake and Physical Activity Levels in Australian University Students. Nutrients.

[36] Husain W., Ashkanani F. (2020). Does COVID-19 change dietary habits and lifestyle behaviours in Kuwait: A community-based cross-sectional study. Environ. Health Prev. Med..

[37] Pellegrini M., Ponzo V., Rosato R., Scumaci E., Goitre I., Benso A., Belcastro S., Crespi C., De Michieli F., Ghigo E. (2020). Changes in Weight and Nutritional Habits in Adults with Obesity during the “Lockdown” Period Caused by the COVID-19 Virus Emergency. Nutrients.

[38] Zachary Z., Brianna F., Brianna L., Garrett P., Jade W., Alyssa D., Mikayla K. (2020). Self-quarantine and weight gain related risk factors during the COVID-19 pandemic. Obes. Res. Clin. Pract..

[39] Marsola C.D.M., Cunha L.M., Carvalho-Ferreira J.P., da Cunha D.T. (2021). A dataset of food choice motives among adults consumers in Brazil: The use of Food Choice Questionnaire. Data Brief.

[40] World Health Organization Food and Nutrition Tips during Self-Quarantine. https://www.euro.who.int/en/health-topics/health-emergencies/coronavirus-covid-19/publications-and-technical-guidance/food-and-nutrition-tips-during-self-quarantine.

[41] Gehlich K.H., Beller J., Lange-Asschenfeldt B., Köcher W., Meinke M.C., Lademann J. (2019). Consumption of fruits and vegetables: Improved physical health, mental health, physical functioning and cognitive health in older adults from 11 European countries. Aging Ment. Health.

[42] Slavin J.L., Lloyd B. (2012). Health Benefits of Fruits and Vegetables. Adv. Nutr. Int. Rev. J..

[43] Collese T.S., Nascimento-Ferreira M.V., De Moraes A.C.F., Rendo-Urteaga T., Bel-Serrat S., Moreno L.A., Carvalho H.B. (2017). Role of fruits and vegetables in adolescent cardiovascular health: A systematic review. Nutr. Rev..

[44] Głąbska D., Guzek D., Groele B., Gutkowska K. (2020). Fruit and Vegetable Intake and Mental Health in Adults: A Systematic Review. Nutrients.

[45] Brookie K.L., Best G.I., Conner T.S. (2018). Intake of Raw Fruits and Vegetables Is Associated with Better Mental Health Than Intake of Processed Fruits and Vegetables. Front. Psychol..

[46] Głąbska D., Guzek D., Groele B., Gutkowska K. (2020). Fruit and vegetables intake in adolescents and mental health: A systematic review. Rocz. Państw. Zakł. Hig..

[47] Ruiz-Roso M.B., de Carvalho Padilha P., Mantilla-Escalante D.C., Ulloa N., Brun P., Acevedo-Correa D., Peres W.A.F., Martorell M., Aires M.T., de Oliveira Cardoso L. (2020). COVID-19 confinement and changes of adolescent’s dietary trends in Italy, Spain, Chile, Colombia and Brazil. Nutrients.

[48] Pietrobelli A., Pecoraro L., Ferruzzi A., Heo M., Faith M., Zoller T., Antoniazzi F., Piacentini G., Fearnbach S.N., Heymsfield S.B. (2020). Effects of COVID-19 Lockdown on Lifestyle Behaviors in Children with Obesity Living in Verona, Italy: A Longitudinal Study. Obesity.

[49] Allabadi H., Dabis J., Aghabekian V., Khader A., Khammash U. (2020). Impact of COVID-19 lockdown on dietary and lifestyle behaviours among adolescents in Palestine. Dyn. Hum. Health.

[50] Bhutani S., Cooper J.A., Vandellen M.R. (2021). Self-reported Changes in Energy Balance Behaviors during COVID-19-related Home Confinement: A Cross-sectional Study. Am. J. Health Behav..

[51] Bracale R., Vaccaro C.M. (2020). Changes in food choice following restrictive measures due to COVID-19. Nutr. Metab. Cardiovasc. Dis..

[52] Di Renzo L., Gualtieri P., Pivari F., Soldati L., Attinà A., Cinelli G., Leggeri C., Caparello G., Barrea L., Scerbo F. (2020). Eating habits and lifestyle changes during COVID-19 lockdown: An Italian survey. J. Transl. Med..

[53] Rodríguez-Pérez C., Molina-Montes E., Verardo V., Artacho R., García-Villanova B., Guerra-Hernández E.J., Ruíz-López M.D. (2020). Changes in Dietary Behaviours during the COVID-19 Outbreak Confinement in the Spanish COVIDiet Study. Nutrients.

[54] Bhutani S., Cooper J.A. (2020). COVID-19-Related Home Confinement in Adults: Weight Gain Risks and Opportunities. Obesity.

[55] Mehta V. The Impact of COVID-19 on the Dietary Habits of Middle-Class Population in Mulund, Mumbai, India. https://preprints.aijr.org/index.php/ap/preprint/view/82.

[56] Shang D., Wu W. (2017). Understanding mobile shopping consumers’ continuance intention. Ind. Manag. Data Syst..

[57] Dirsehan T., Cankat E. (2021). Role of mobile food-ordering applications in developing restaurants’ brand satisfaction and loyalty in the pandemic period. J. Retail. Consum. Serv..

[58] Mortimer G., Hasan S.F.E., Andrews L., Martin J. (2016). Online grocery shopping: The impact of shopping frequency on perceived risk. Int. Rev. Retail. Distrib. Consum. Res..

[59] Driediger F., Bhatiasevi V. (2019). Online grocery shopping in Thailand: Consumer acceptance and usage behavior. J. Retail. Consum. Serv..

[60] Sreeram A., Kesharwani A., Desai S. (2017). Factors affecting satisfaction and loyalty in online grocery shopping: An integrated model. J. Indian Bus. Res..

[61] Huang Y., Oppewal H. (2006). Why consumers hesitate to shop online: An experimental choice analysis of grocery shopping and the role of delivery fees. Int. J. Retail Distrib. Manag..

[62] Morganosky M.A., Cude B.J. (2000). Consumer response to online grocery shopping. Int. J. Retail Distrib. Manag..

[63] Shankar V., Smith A.K., Rangaswamy A. (2003). Customer satisfaction and loyalty in online and offline environments. Int. J. Res. Mark..

[64] Chu J., Arce-Urriza M., Cebollada-Calvo J.-J., Chintagunta P.K. (2010). An Empirical Analysis of Shopping Behavior Across Online and Offline Channels for Grocery Products: The Moderating Effects of Household and Product Characteristics. J. Interact. Mark..

[65] Statista (2020). Online Food Delivery.

[66] Long N.N., Khoi B.H. (2020). An Empirical Study about the Intention to Hoard Food during COVID-19 Pandemic. Eurasia J. Math. Sci. Technol. Educ..

[67] Lyson T.A., Raymer A.L. (2000). Stalking the wily multinational: Power and control in the US food system. Agric. Hum. Values.

[68] Alexander E., Yach D., Mensah G.A. (2011). Major multinational food and beverage companies and informal sector contributions to global food consumption: Implications for nutrition policy. Glob. Health.

[69] Alfranca O., Rama R., Von Tunzelmann N. (2003). Technological fields and concentration of innovation among food and beverage multinationals. Int. Food Agribus. Manag. Rev..

[70] Yu J. Chinese Fresh Food Sales Boosted by COVID-19. https://www.kantar.com/north-america/inspiration/coronavirus/chinese-fresh-food-sales-boosted-by-covid-19.

[71] Bloomberg News Chinese Kids are Drinking More Milk to Boost Immune System after Covid. https://theprint.in/health/chinese-kids-are-drinking-more-milk-to-boost-immune-system-after-covid/467198/.

[72] Xinhua Eating Habit Changes among Chinese during COVID-19 Epidemic. https://www.chinadaily.com.cn/a/202008/20/WS5f3e3107a310834817261a8c.html.

[73] Carroll N., Sadowski A., Laila A., Hruska V., Nixon M., Ma D.W., Haines J.J., on behalf of the Guelph Family Health Study (2020). The Impact of COVID-19 on Health Behavior, Stress, Financial and Food Security among Middle to High Income Canadian Families with Young Children. Nutrients.

[74] Giacalone D., Frøst M.B., Rodríguez-Pérez C. (2020). Reported Changes in Dietary Habits During the COVID-19 Lockdown in the Danish Population: The Danish COVIDiet Study. Front. Nutrients.

[75] Sidor A., Rzymski P. (2020). Dietary Choices and Habits during COVID-19 Lockdown: Experience from Poland. Nutrients.

[76] Buckland N.J., Swinnerton L.F., Ng K., Price M., Wilkinson L.L., Myers A., Dalton M. (2020). Susceptibility to increased high energy dense sweet and savoury food intake in response to the COVID-19 lockdown: The role of craving control and acceptance coping strategies. Appetite.

[77] Poelman M.P., Gillebaart M., Schlinkert C., Dijkstra S.C., Derksen E., Mensink F., Hermans R.C., Aardening P., de Ridder D., de Vet E. (2021). Eating behavior and food purchases during the COVID-19 lockdown: A cross-sectional study among adults in the Netherlands. Appetite.

[78] Hirvonen K., de Brauw A., Abate G.T. (2021). Food Consumption and Food Security during the COVID-19 Pandemic in Addis Ababa. Am. J. Agric. Econ..

[79] United Nations in China UN Helps China Reduce Food Waste to Meet SDGs. http://cn.un.org.cn/info/6/649.html.

[80] UN Environment Programme SDG 12.3 Food Waste Index. https://www.unep.org/thinkeatsave/about/sdg-123-food-waste-index#:~:text=SDG%2012.3%3A%20%22By%202030%2C,including%20post%2Dharvest%20losses.%22&text=SDG%20Target%2012.3%20seeks%20to,loss%20during%20production%20and%20supply.

[81] Barclay A.W., Brand-Miller J. (2011). The Australian Paradox: A Substantial Decline in Sugars Intake over the Same Timeframe That Overweight and Obesity Have Increased. Nutrients.

[82] Czoli C.D., Jones A.C., Hammond D. (2019). Trends in sugary drinks in Canada, 2004 to 2015: A comparison of market sales and dietary intake data. Public Health Nutr..

[83] Castaldelli-Maia J.M., Segura L.E., Martins S.S. (2021). The concerning increasing trend of alcohol beverage sales in the U.S. during the COVID-19 pandemic. Alcohol.

[84] Development and Reform System Price Monitoring Agency Daily Monitoring of Major Food Prices in the Region during the Period of Pandemic Prevention and Control. http://fgw.nmg.gov.cn/sytpzs/202103/t20210326_1316773.html.

[85] Chen J., Yang C.C. (2021). The impact of the National Nutrition Program 2017–2030 on people’s food purchases: A revenue-based perspective. Nutrients.

[86] He L.J., Chen J. (2021). Does Mandatory Audit Partner Rotation Influence Auditor Selection Strategies?. Sustainability.

[87] Yang C.C., Chen J.X., Yang W.C. (2020). The Impact of the Amendment of Taiwan’s Certified Public Accountant Act in 2007 on Large Accounting Firms. Sustainability.

[88] Chen J., Yang C.C. (2021). Competitive Revenue Strategies in the Medical Consumables Industry: Evidence from Human Resources, Research and Development Expenses and Industry Life Cycle. Int. J. Environ. Res. Public Health.

[89] Belitski M., Guenther C., Kritikos A.S., Thurik R. (2021). Economic effects of the COVID-19 pandemic on entrepreneurship and small businesses. Small Bus. Econ..

[90] International Labour Organization (ILO) ILO Monitor: COVID-19 and the World of Work. https://www.ilo.org/wcmsp5/groups/public/@dgreports/@dcomm/documents/briefingnote/wcms_749399.pdf.

[91] Tesfaye A., Habte Y., Minten B. The Quest for Safer Foods: The COVID-19 Crisis and Dairy Value Chains in Ethiopia. https://essp.ifpri.info/2020/05/12/the-quest-for-safer-foods-the-covid-19-crisis-and-dairy-value-chains-in-ethiopia/.

[92] Ceballos F., Hernandez M.A., Paz C. (2021). Short-term impacts of COVID-19 on food security and nutrition in rural Guatemala: Phone-based farm household survey evidence. Agric. Econ..

[93] Di Crosta A., Ceccato I., Marchetti D., La Malva P., Maiella R., Cannito L., Cipi M., Mammarella N., Palumbo R., Verrocchio M.C. (2021). Psychological factors and consumer behavior during the COVID-19 pandemic. PLoS ONE.

[94] Johnny H., Hui D., Kim A., Zhang Y. Cautiously Optimistic: Chinese Consumer Behavior Post-COVID-19. https://www.mckinsey.com/industries/consumer-packaged-goods/our-insights/cautiously-optimistic-chinese-consumer-behavior-post-covid-19#:~:text=Chinese%20consumers%20are%20gradually%20regaining,latest%20survey%20of%20consumer%20attitudes.

[95] European Commission About Food Waste. https://ec.europa.eu/food/safety/food_waste_en.

[96] Willett W., Rockström J., Loken B., Springmann M., Lang T., Vermeulen S., Garnett T., Tilman D., DeClerck F., Wood A. (2019). Food in the Anthropocene: The EAT–Lancet Commission on healthy diets from sustainable food systems. Lancet.

[97] Chandrasekaran B., Ganesan T.B. (2021). Sedentarism and chronic disease risk in COVID 19 lockdown—A scoping review. Scott. Med. J..

[98] Oriental Fortune, Zhongjing Foods: The Company’s “2021 Annual Report” Is Scheduled to Be Disclosed on April 23, 2022. http://finance.eastmoney.com/a/202201132246991381.html.

[99] Daily Economic News, Joyvio Foods: The Company Will Disclose Its 2021 Annual Report on April 8, 2022. http://www.nbd.com.cn/articles/2022-02-10/2119986.html.

[100] Oriental Fortune, Sanquan Food: The Annual Report Appointment Disclosure Time Is April 15. https://finance.eastmoney.com/a/202104011869088858.html.

